# Two subtypes of colorectal tumor with distinct molecular features in familial adenomatous polyposis

**DOI:** 10.18632/oncotarget.11510

**Published:** 2016-08-22

**Authors:** Kiyoko Takane, Keisuke Matsusaka, Satoshi Ota, Masaki Fukuyo, Yao Yue, Motoi Nishimura, Eiji Sakai, Kazuyuki Matsushita, Hideaki Miyauchi, Hiroyuki Aburatani, Yukio Nakatani, Tadatoshi Takayama, Hisahiro Matsubara, Kiwamu Akagi, Atsushi Kaneda

**Affiliations:** ^1^ Department of Molecular Oncology, Graduate School of Medicine, Chiba University, Chiba, Japan; ^2^ Department of Digestive Surgery, Nihon University School of Medicine, Tokyo, Japan; ^3^ Department of Pathology, Chiba University Hospital, Chiba, Japan; ^4^ Department of Molecular Diagnosis, Graduate School of Medicine, Chiba University, Chiba, Japan; ^5^ Department of Frontier Surgery, Graduate School of Medicine, Chiba University, Chiba, Japan; ^6^ Department of Diagnostic Pathology, Graduate School of Medicine, Chiba University, Chiba, Japan; ^7^ Department of Gastroenterology, Yokohama City University School of Medicine, Yokohama, Japan; ^8^ Genome Science Division, Research Center for Advanced Science and Technology, The University of Tokyo, Tokyo, Japan; ^9^ Division of Molecular Diagnosis, Saitama Cancer Center, Saitama, Japan

**Keywords:** CIMP, colorectal cancer, DNA methylation, familial adenomatous polyposis (FAP), KRAS

## Abstract

While sporadic colorectal cancer (CRC) is classified into several molecular subtypes, stratification of familial colorectal tumors is yet to be well investigated. We previously established two groups of methylation markers through genome-wide DNA methylation analysis, which classified sporadic CRC and adenoma into three distinct subgroups: high-, intermediate-, and low-methylation epigenotypes. Here, we investigated familial adenomatous polyposis (FAP), through quantitative methylation analysis of 127 samples (16 cancers, 96 adenomas, and 15 benign mucosa from 14 patients with FAP) using six Group-1 and 14 Group-2 methylation markers, *APC*, *BRAF*, and *KRAS* mutation analysis, and CTNNB1 and TP53 immunohistochemical analysis. All the 14 patients presented with *APC* germline mutation. Three were from the same family and presented the same *APC* mutation. FAP tumors lacked *BRAF*-mutation(+) high-methylation epigenotype and were classified into two methylation epigenotypes. While 24 of 112 tumor samples showed intermediate-methylation epigenotype significantly correlating with *KRAS*-mutation(+) (*P*=3×10^-4^), 88 tumor samples showed low-methylation epigenotype correlating with the absence of *KRAS*- and *BRAF*-mutations. Similar to sporadic CRC, CTNNB1 was frequently activated at the adenoma stage, and *TP53* mutation occurred during cancer development from adenoma. Whereas some patients showed a single epigenotype in all tumors throughout the colon, tumors with two distinct epigenotypes developed within a family with the same *APC* mutation or even within one patient. Methylation accumulation significantly correlated with proximal location and older age. These results indicate that there are at least two distinct molecular subtypes of FAP tumors, resembling sporadic CRC and independent from the *APC* germline mutation status.

## INTRODUCTION

Colorectal cancer (CRC) arises because of the accumulation of epigenetic and genetic alterations [1-3]. Gene mutations of *KRAS*, *APC*, and *TP53* are well-known genetic alterations, which were demonstrated in the model of adenoma-carcinoma sequence [4]. Recent exome sequencing studies of CRC revealed the involvement of somatic mutation of other genes, e.g., *SOX9*, *SMAD4*, *PIK3CA*, *ARID1A*, and *NRAS* [5-7]. According to a report by the Cancer Genome Atlas (TCGA), CRC is classified into hypermutated and non-hypermutated CRC, and hypermutated CRC exhibits frequent gene mutations such as *BRAF* and *MSH6*, microsatellite instability, and promoter methylation of *MLH1* [6]. Aberrant DNA methylation of promoter CpG islands has been reported as one of the most important epigenomic alterations in CRC [8, 9]. The CRC subtype with frequent aberrant methylation, so-called CpG island methylator phenotype (CIMP) [10, 11], overlaps with the hypermutated CRC [6].

We and others previously performed epigenotyping of CRC, using comprehensive and quantitative DNA methylation data [12-14]. Two groups of methylation marker genes were established to clearly classify CRC into three distinct epigenotypes [12]. High-methylation epigenotype (or CIMP) showed methylation of both Group-1 and Group-2 markers, while intermediate-methylation epigenotype showed methylation of Group-2, but not of Group-1 markers, and low-methylation epigenotype showed methylation of neither Group-1 nor Group-2 markers. High- and intermediate-methylation epigenotypes strongly correlated with *BRAF* and *KRAS* mutations, respectively, and low-methylation epigenotype correlated with the absence of these oncogene mutations, suggesting the existence of at least three distinct pathways in the genesis of CRC.

Familial adenomatous polyposis (FAP) and Lynch syndrome (also known as hereditary nonpolyposis CRC) are the two major autosomal dominant forms of heritable CRC, which accounts for 5-15% of all CRC cases [15-17]. Lynch syndrome can be caused by mutations in the mismatch repair genes, e.g., *MLH1*, *MSH2*, *MSH6*, and *PMS2*, and is inherited in an autosomal dominant manner. For FAP, *APC* germline mutation is known to be the cause for colonic polyps. *APC* is a tumor suppressor gene that is responsible for regulating the *Wnt* signaling pathway; while one allele was inactivated by germline mutation, the other allele is involved with loss of heterozygosity at 50-59% or another mutation at 33% [18, 19]. Frequent mutations of *KRAS* (36-44%) [20, 21] and *TP53* (31-40%) [22, 23] were reportedly involved in FAP cancer, while mutation frequencies of those in adenomas are rather low, 6-36% for *KRAS* [20, 24, 25] and 5-38% for *TP53* [22, 23, 26]. In spite of the extremely high risk of cancer incidence in FAP, the molecular basis of tumorigenesis in FAP has not been fully investigated. The ‘second hit’ against *APC* was not necessarily identified in *APC*-mutation(+) FAP tumors [19]. Approximately 20% of patients with FAP do not possess *APC* germline mutation, and responsible [27] [24].

In this study, we analyzed epigenetic and genetic features of FAP tumors. Using quantitative DNA methylation data, we determined that there are at least two molecular subtypes in FAP tumors, which resembled sporadic CRC: intermediate-methylation epigenotype with *KRAS* mutation and low-methylation epigenotype with no oncogene mutation. While some patients showed a single epigenotype in all tumors throughout the colon, tumors with two distinct epigenotypes developed within a family with the same *APC* mutation or even within one patient. These results indicate that there are at least two distinct molecular subtypes in FAP tumors, resembling sporadic CRC and independent from *APC* germline mutation status. Methylation accumulation might be causally affected by environmental factors, e.g., proximal location and aging.

## RESULTS

### Mutation analysis of BRAF and KRAS and immunostaining of CTNNB1 and TP53

**Figure 1:**
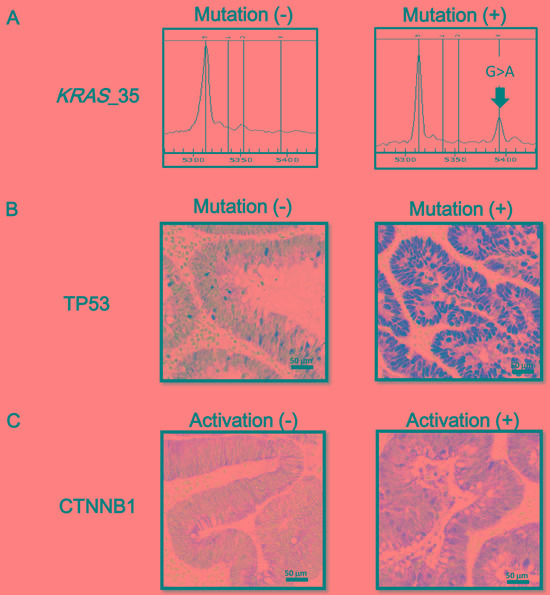
Oncogene mutation status and immunostaining of TP53 and CTNNB1 **A.** Mutation at nucleotide 35 of the *KRAS* gene (G > A) is representatively shown. *BRAF* mutation was not detected in any FAP tumor samples. **B.** Immunostaining of TP53. When nuclear staining was present in tumor cells, TP53 mutation was considered as positive. **C.** Immunostaining of CTNNB1. A case with cellular membrane staining, i.e. CTNNB1-activation(-) (*left*), and a case with nuclear and cytoplasmic staining, i.e. CTNNB1-activation(+) (*right*), are representatively shown.

While *KRAS* mutations were frequently detected in 46 (41%) out of 112 FAP tumor samples, no sample was *BRAF*-mutation(+) (Figure 1). We performed TP53 and CTNNB1 immunostaining for 86 samples, 14 (16%) were regarded as *TP53*-mutation(+), and 46 (53%) were regarded as CTNNB1-activation(+).

### Quantitative DNA methylation analysis

**Figure 2:**
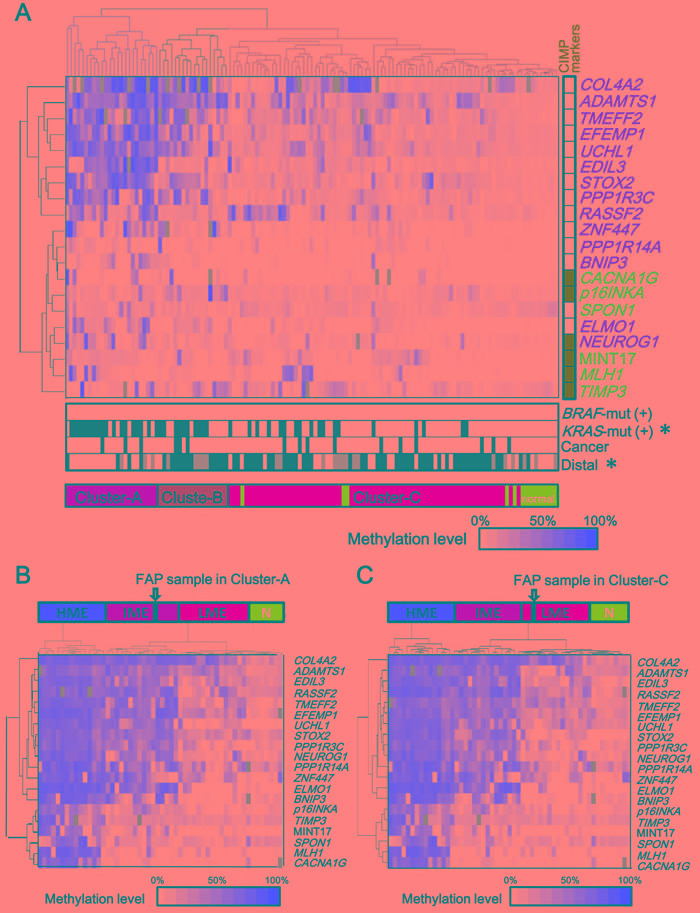
Two epigenotypes of FAP tumors **A.** Methylation levels of 6 Group-1 markers (*blue*) including *p16INKA*, *TIMP3*, *SPON1*, MINT17, *MLH1*, and *CACNA1G* and 14 Group-2 markers (*orange*) including *ADAMTS1*, *TMEFF2*, *STOX2*, *COLA4A2*, *EDIL3*, *UCHL1*, *RASSF2*, *ELMO1*, *PPP1R3C*, *PPP1R14A*, *BNIP3*, *ZNF447*, and *NEUROG1* are shown for each tumor or normal sample of FAP cases, and unsupervised hierarchical clustering analysis was performed. Methylation was quantitatively analyzed by pyrosequencing and shown in color scale, or by *grey box* when not analyzed. FAP samples were clearly classified into clusters: Cluster-A with higher methylation and Cluster-C with lower methylation. The 24 tumors in Cluster-A showed high methylation of Group-2 markers, but low methylation of Group-1 markers, and correlated with *KRAS*-mutation(+) (*P* = 1×10^-4^). The 70 tumors and 15 normal samples in Cluster-C showed low methylation of both Group-1 and Group-2 markers. *CIMP markers*: Classical CIMP markers [11, 13, 29, 30] are shown in green. *BRAF-mut(+)* *or KRAS-mut(+)*: Samples positive for *BRAF*-mutation or *KRAS*-mutation are shown in *black*. *Cancer*: Cancer is shown in *black* and adenoma in *white*. *Distal*: Tumors in the distal colon are shown in *black*, those in the proximal colon in *white*, and those in an unknown location in *grey*. **B.** and **C.** Hierarchical clustering of FAP sample with previously analyzed sporadic CRC samples. The methylation epigenotype of each FAP sample was evaluated by unsupervised hierarchical clustering analysis with 45 sporadic CRC samples including 15 high-, 15 intermediate-, and 15 low-methylation epigenotypes. The methylation epigenotype of the 24 tumors in Cluster-A was considered as the intermediate-methylation epigenotype by hierarchical clustering analysis with 45 CRC samples (B). The methylation epigenotype of the 85 samples in Cluster-C was considered as the low-methylation epigenotype (C). Cluster-A and Cluster-C both included cancer samples, without a significant difference in frequency (3/24 *vs*. 10/70, *P* = 0.5). Two of the 18 samples in Cluster-B were clustered with intermediate-methylation CRC samples, whereas the other 16 samples in Cluster-B were clustered with low-methylation CRC samples.

Using quantitative methylation data obtained from pyrosequencing, we performed hierarchical clustering on 127 colorectal samples of FAP cases including 112 malignant and 15 benign mucosa samples (Figure 2A). These samples were clearly classified into clusters: Cluster-A (*n* = 24) with higher methylation and Cluster-C (*n* = 70) with lower methylation. The 24 tumor samples in Cluster-A significantly correlated with the presence of *KRAS* mutation (*P* = 1×10^-4^), and proximal location (*P* = 3×10^-6^) (Figure 2A). To evaluate methylation epigenotype of this cluster by comparison with the previously established methylation epigenotypes of sporadic CRC [12, 28], their methylation status was examined with 45 sporadic CRC samples, including 15 high-, 15 intermediate-, and 15 low-methylation epigenotypes, which had been previously evaluated [12]. Hierarchical clustering analysis revealed that all 24 tumor samples in Cluster-A were clustered into intermediate-methylation epigenotype (Figure 2B). The 70 malignant and 15 benign mucosa samples in Cluster-C significantly correlated with the absence of *KRAS* mutation and distal location (Figure 2A). These were also compared with the 45 sporadic CRC samples, revealing that all 85 samples were clustered into the low-methylation epigenotype (Figure 2C).

Adenocarcinoma samples were detected in both Cluster-A and Cluster-C, without statistical significance (3/24 *vs*. 10/70, *P* = 0.5). There were 18 samples in Cluster-B among the 127 FAP samples (Figure 2A), and the hierarchical clustering analysis with the 45 CRC samples showed that two were classified into intermediate-methylation epigenotype and 16 were low-methylation epigenotype.

None of the FAP tumors showed high-methylation epigenotype. Considering that no *BRAF*-mutation was detected, it suggested that high-methylation epigenotype with *BRAF* mutation is not involved in tumorigenesis of FAP tumor. Six classical CIMP markers proposed by Issa et al. or Laird et al., i.e. *CACNA1G* (also known as MINT31), *p16INKA*, *NEUROG1*, MINT17, *MLH1*, and *TIMP3* [11, 13, 29, 30], were analyzed in this study, and all of these markers were mostly unmethylated in FAP tumors, indicating CIMP(-).

**Figure 3:**
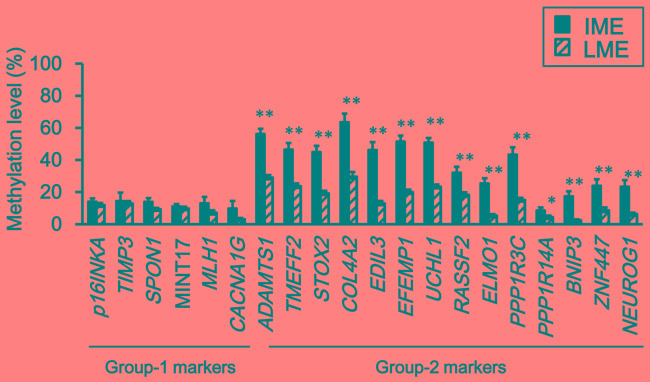
Comparison of methylation levels between intermediate- and low-methylation epigenotypes Methylation levels are represented by means ± standard errors. *IME*, intermediate-methylation epigenotype. *LME*, low-methylation epigenotype. All Group-2 markers showed significantly higher methylation levels in intermediate-methylation samples than in low-methylation samples (*P < 0.05, **P < 0.01, Student's *t*-test). Methylation levels of the six Group-1 markers were low in both intermediate- and low-methylation samples, and there was no significant difference between the two epigenotypes.

To confirm that the difference of intermediate- and low-methylation epigenotypes in FAP tumors was due to differences in the methylation levels in Group-2 markers, which is feature of sporadic CRC [12, 28], their methylation levels in intermediate- and low-methylation tumors were compared (Figure 3). While all Group-1 markers (≅ CIMP markers) showed low methylation levels in both intermediate- and low-methylation tumors, all Group-2 markers showed significantly higher methylation levels in intermediate-methylation samples when compared to the low-methylation samples.

### Comparison between adenoma and cancer

**Figure 4:**
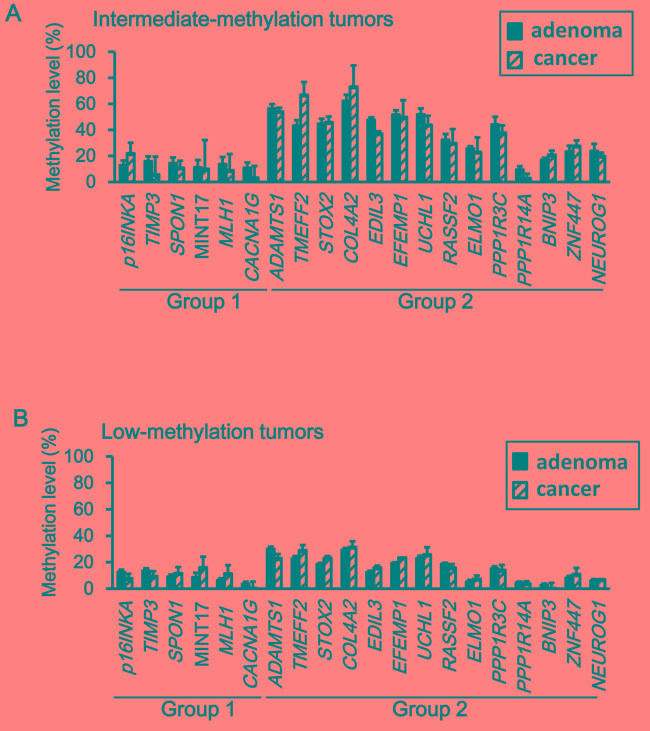
Comparison of methylation levels between adenoma and cancer Neither intermediate-methylation tumors (**A)** nor low-methylation tumors (**B)** did show a significant increase of the methylation level from adenoma to cancer samples (*P* < 0.05, Student's *t*-test).

Next, methylation levels of individual genes were compared between adenoma and cancer. None of the genes showed a significant increase of methylation level in cancer when compared with adenoma (Figure 4).

**Figure 5:**
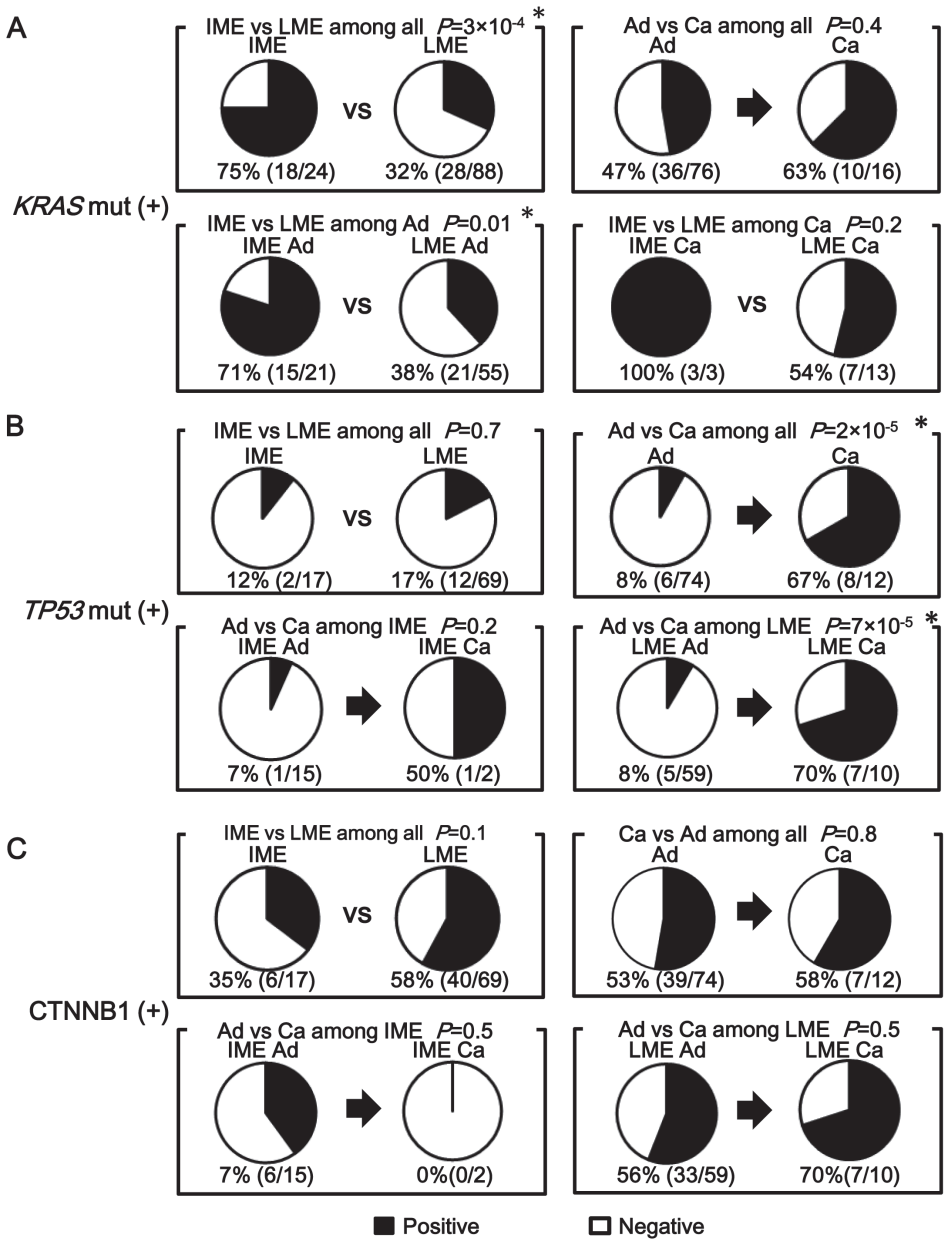
Comparison of *KRAS* mutation, *TP53* mutation, and CTNNB1 activation between adenoma and cancer, and between low- and intermediate-methylation epigenotypes **A.** The frequency of *KRAS*-mutation(+). A significant correlation between intermediate-methylation and *KRAS* mutation was detected when analyzing all the FAP tumors (*P* = 3×10^-4^). This correlation was aleady detected at the adenoma stage (*P* = 0.01). The frequency of *KRAS*-mutation(+) did not significantly increase from adenoma to cancer (*P* = 0.4). **B.** The frequency of *TP53* mutation. The frequency of *TP53* mutation significantly increased in cancer compared with adenoma (*P* = 2×10^-5^). Among low-methylation tumors, cancer showed significantly higher frequency of *TP53* mutation compared with adenoma (*P* = 7×10^-5^). **C.** The frequency of CTNNB1-activation(+). Frequent CTNNB1 activation was observed in both adenoma (39/74) and cancer (7/12) with no significant difference (*P* = 0.8). Among low-methylation tumors, CTNNB1 activation was frequently observed in both adenoma (33/59) and cancer (7/10) with no significant difference (*P* = 0.5).

The frequency of *KRAS*-mutation(+) did not increase significantly in cancer compared with that in adenoma and a significant correlation between intermediate-methylation and *KRAS*-mutation(+) was already detected at the adenoma stage (Figure 5A). As detected in intermediate-methylation sporadic CRC [12, 28, 31], it suggested that methylation accumulation and *KRAS*-mutation(+) are mostly completed by the adenoma stage.

The frequency of *TP53*-mutation(+) increased in cancer compared with that in adenoma (*P* = 2×10^-5^). Low-methylation cancer showed significantly higher frequency of *TP53*-mutation(+) than low-methylation adenoma (7/10 *vs*. 5/59, *P* = 7×10^-5^) (Figure 5B).

For activation of CTNNB1, 33 (56%) of 59 adenoma samples were CTNNB1-activation(+) whereas seven (70%) of 10 cancer samples were CTNNB1-activation(+) in low-methylation FAP samples, showing frequent activation in both adenoma and cancer with no significant difference (*P* = 0.5) (Figure 5C).

### Methylation epigenotypes in each FAP case

**Figure 6:**
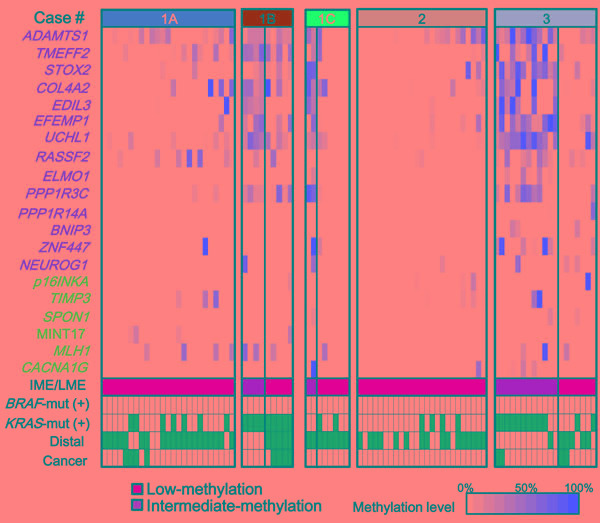
Methylation patterns of tumors in each patient Case-1A and Case-2 showed single epigenotype in all the tumors. Case-1B, Case-1C, and Case-3 showed two distinct epigenotypes in a single patient. Case-1A, Case-1B and Case-1C were from the same family and possessed the same *APC* germline mutation (L540X). Nevertheless, Case-1B and Case-1C showed two epigenotypes and Case-1A showed low-methylation epigenotype only throughout the colon. For Case-3, intermediate-methylation tumors were preferentially observed with *KRAS* mutation (*P* = 0.03) and in the proximal colon (*P* = 0.009). For Case-1B, a significant association was observed between intermediate-methylation epigenotype and proximal location (*P* = 0.007).

Among the 14 FAP cases, > 15 tumor samples were analyzed in three cases, Case-1A, Case-2, and Case-3. When we analyzed methylation levels of tumors in these FAP cases individually, methylation patterns of tumors showed interesting tendencies (Figure 6).

First, while all the tumors in Case-1A and Case-2 showed low-methylation epigenotype, Case-3 developed both low- and intermediate-methylation tumors. If we compare Case-1A with two other patients from the same family (Cases-1B and 1C), the two patients also developed both low- and intermediate-methylation tumors, although only nine and eight tumors were analyzed (Figure 6). A single FAP patient did not necessarily develop a single methylation epigenotype.

Secondly, the two methylation epigenotypes could occur independently of *APC* mutation status. Case-3 showed both low- and intermediate-methylation tumors within a single patient. Interestingly, intermediate-methylation epigenotype significantly correlated with *KRAS* mutation (10 of 12, compared with two of seven in low-methylation, *P* = 0.03).

Thirdly, the methylation status might perhaps correlate with tumor location. Proximal location was significantly associated with intermediate-methylation epigenotype in Case-3 (*P* = 0.009). Although the number of tumors was small, this significant association was also detected in Case-1B (*P* = 0.007)

### Comparison using linear single regression

**Figure 7:**
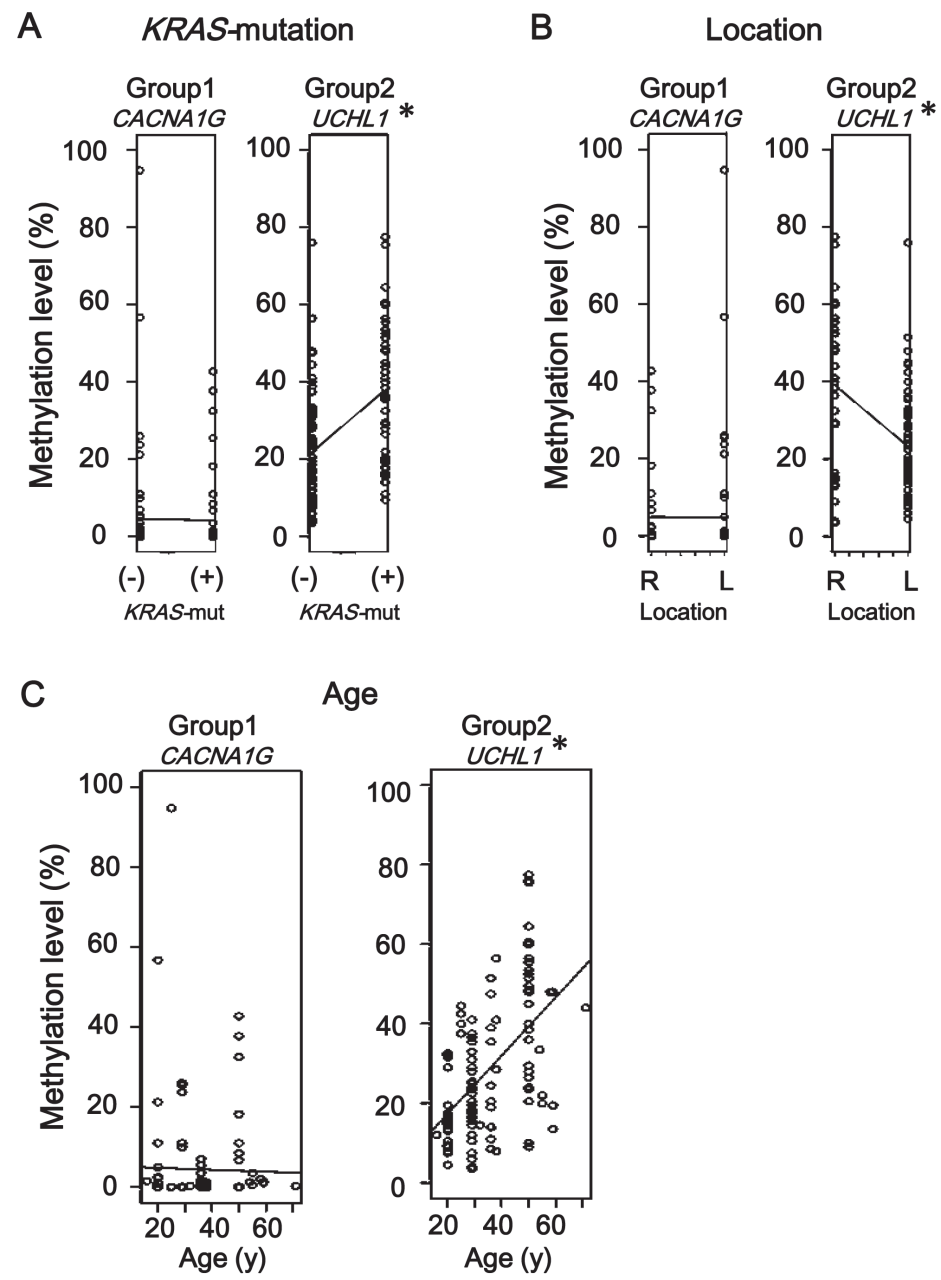
Comparison using linear single regression model **A.** Association of methylation accumulation with *KRAS*-mutation. Eight of 14 Group-2 markers, e.g., *UCHL1*, showed significant correlation between higher methylation level and *KRAS*-mutation(+) status, none of Group-1 markers did, e.g., *CACNA1G*. (*See* Supplementary Figure S1) **B.** Association of methylation accumulation with location. Five of 14 Group-2 markers, e.g., *UCHL1*, showed significant correlation between higher methylation level and proximal location, while none of Group-1 markers did, e.g., *CACNA1G*. (*See* Supplementary Figure S2) **C.** Association of methylation accumulation with age. Twelve of 14 Group-2 markers, e.g., *UCHL1*, showed a significant correlation between higher methylation level and age, while none of Group-1 markers did, e.g. *CACNA1G.* (*See* Supplementary Figure S3) Since six Group-1 markers and 14 Group-2 markers were evaluated for each factor, *P*-value < 0.008 (i.e., 0.05/6) and < 0.004 (i.e., 0.05/14) were considered significant, respectively, instead of *P* < 0.05 (*).

To evaluate the possible association of methylation accumulation with tumor location and with age, methylation levels and these factors as well as *KRAS* mutation status were analyzed by linear single regression model using all samples (Figure 7). Eight Group-2 markers showed a significant correlation between higher methylation level and *KRAS*-mutation(+), while none of Group-1 markers did (Figure 7A and Supplementary Figure S1). Five of Group-2 markers, *EDIL3*, *EFEMP1*, *UCHL1*, *ELMO1*, and *BNIP3*, showed a significant correlation between higher methylation level and proximal location, while none of Group-1 markers did (Figure 7B and Supplementary Figure S2). Twelve of Group-2 markers showed a significant correlation between higher methylation level and age, while none of Group-1 markers did (Figure 7C and Supplementary Figure S3).

## DISCUSSION

In this study, we stratified FAP tumors into distinct molecular subtypes. Quantitative DNA methylation analysis of Group-1 and Group-2 markers indicated that there are at least two subtypes in FAP tumors: low- and intermediate-methylation epigenotypes. As observed in sporadic CRC and adenoma, intermediate-methylation epigenotype is accompanied with *KRAS* mutation and low-methylation epigenotype is accompanied with no oncogene mutation. These subtypes might be formed independently from *APC* germline mutation status, and both subtypes could develop malignant tumors.

There have been few studies reporting aberrant DNA methylation in FAP tumors. Wynter *et al.* analyzed eight methylation markers (MINT1, MINT2, MINT12, and MINT31 markers, and promoter regions of *HPP1*, *MGMT*, *p14*, and *p16*) in sporadic and FAP adenoma samples and suggested that FAP adenoma might develop through non-CIMP pathway [24]. Whereas CIMP(+)/high-methylation tumors could be distinguished from CIMP(-) tumors using the classic CIMP markers (≅Group-1 markers), two groups of markers should be necessary to distinguish three subtypes, including low- and intermediate-methylation epigenotypes in CIMP(-) tumors [12-14]. In the present study, we analyzed both Group-1 and Group-2 markers quantitatively and clearly demonstrated that, while high-methylation epigenotype with *BRAF* mutation is not involved, there are at least two molecular subtypes of FAP tumors: low- and intermediate-methylation epigenotypes. The hierarchical clustering analysis using FAP tumor samples (Figure 2), however, showed three clusters: Cluster-A, Cluster-B, and Cluster-C. Although Cluster-A is considered equivalent to the intermediate-methylation epigenotype and Cluster-C equivalent to the low-methylation epigenotype in sporadic CRC, further studies using more samples and methylation markers are necessary to determine if FAP tumors consist of these two epigenotypes only or if there are other distinct epigenotypes (e.g., Cluster-B), and whether there are any specific and suitable methylation markers to stratify FAP tumors.

*KRAS* mutation has been frequently detected [20, 21, 24, 25] in adenoma and cancer in FAP patients. In agreement with these previous reports, *KRAS* mutation was frequently detected in 46 (41%) out of 112 tumor samples. However, *KRAS* mutation was preferentially detected in intermediate-methylation epigenotype (*P* = 3×10^-4^) (Figure 5A). The frequency of *KRAS* mutation was not different between adenoma and cancer. Methylation levels did not significantly increase from adenoma to cancer either, suggesting that methylation accumulation to form intermediate-methylation epigenotype and *KRAS* mutation was mostly completed by the adenoma stage, which resembles sporadic colorectal tumors [28, 31].

On the other hand, the frequency of *TP53* mutation was not different between intermediate- and low-methylation epigenotypes, but the frequency of *TP53*-mutation increased in cancer when compared to that in adenoma (*P* = 2×10^-5^) (Figure 5B). *TP53* mutation is necessary during cancer development from adenoma, in agreement with the previously reported concept of adenoma-carcinoma sequence [4, 28, 32]. Our results suggest that *KRAS* mutation occurs preferentially in intermediate-methylation epigenotype and by adenoma stage, and that *TP53* mutation occurs at later stages.

Similarity to sporadic CRC was also detected in CTNNB1 activation. CTNNB1 activation was frequently observed in both intermediate- and low-methylation epigenotypes without statistical significance (*P* = 0.1), and its frequency did not increase from adenoma to cancer (*P* = 0.8) (Figure 5C). This is also in agreement with the adenoma-carcinoma sequence [4, 28, 32].

In addition to the presence of two methylation epigenotypes in FAP tumors, this study revealed that these two types of tumors could be developed within a family with the same *APC* mutation or even within a single FAP patient (Figure 6). Cases-1A, 1B, and 1C were from the same family and possessed the same *APC* germline mutation (L540X) (Table 1). Low-methylation epigenotype was formed throughout the colon in Case-1A, whereas both epigenotypes of tumors were detected in Cases-1B and 1C. The two distinct epigenotypes were also observed in Case-3. These results suggest that the mechanisms underlying the formation of these distinct molecular subtypes should be independent from *APC* germline mutation status.

**Table 1:** Clinical characteristics of FAP patients

Case #	Sex	Age (years)	polyps > 100	mutation of *APC*	# of analyzed adenoma	# of analyzed cancer	# of tumors in each epigenotype	
							LME	IME
1A	Male	29	Yes	L540X	21	4	25	0
1B	Female	50	Yes	L540X	5	4	5	4
1C	Male	25	Yes	L540X	8	0	6	2
2	Male	20	Yes	R564X	25	0	25	0
3	Female	50	Yes	R216X	15	4	7	12
4	Male	36	Yes	R499X	11	0	9	2
5	Female	38	Yes	V1414X	5	0	4	1
6	Female	59	-	R805X	2	1	2	1
7	Female	55	Yes	H1329X	1	1	2	0
8	Male	54	Yes	S1163X	1	0	1	0
9	Male	58	Yes	R332X	1	0	0	1
10	Male	16	Yes	L1385X	0	1	1	0
11	Female	32	Yes	S338X	0	1	1	0
12	Female	71	-	R1114X	1	0	0	1

Case-1A, Case-1B and Case-1C were from the same family, and possessed the same *APC* germline mutation. *LME*, low-methylation epigenotype. *IME*, intermediate-methylation epigenotype.

Whether known environmental factors of methylation accumulation in sporadic CRC could be associated with methylation in FAP tumors remains to be clarified. Aging is known as one of the factors causing aberrant promoter methylation e.g., *ER* and *c-FOS* [33, 34], and aberrant methylation was reported to be preferentially accumulated in the proximal colon [35, 36]. While Group-1 marker methylation was not involved in the development of FAP tumors, 12/14 Group-2 markers showed significant correlation between higher methylation level and older age in the tumorigenesis of FAP (Figure 7). As for location, higher methylation level significantly correlated with proximal location in 5/14 Group-2 markers. These results may suggest that methylation accumulation might occur preferentially in relation to proximal location and older age, but further investigation using more FAP cases and samples is necessary to clarify the contribution of environmental factors to methylation accumulation in patients with FAP.

In summary, there are at least two molecular subtypes in FAP tumors: low- and intermediate-methylation epigenotypes. These subtypes are independent from *APC* germline mutation status, and the both subtypes could develop malignant tumors. Similar to sporadic CRC, *KRAS* mutation significantly correlates with intermediate-methylation epigenotype. CTNNB1 activation and *KRAS* mutation occur at an earlier stage of adenoma formation, and *TP53* occurs at a later stage of FAP tumorigenesis from adenoma to cancer.

## MATERIALS AND METHODS

### Clinical samples

In total, 127 colorectal samples (96 adenoma, 16 cancer, and 15 benign mucosa samples) were obtained from 14 patients with FAP who underwent operation at Chiba University Hospital and Saitama Cancer Center with written informed consents (Table 1). Cases-1A, 1B and 1C were from the same family. Colorectal samples of Cases-1A, 1B, 1C, Case-2, and Case-3 were fixed with 10% formalin and then embedded in paraffin. Samples of Case-4 through Case-12 were frozen immediately after surgical resection and kept at -80 °C until DNA extraction. The formalin-fixed paraffin-embedded (FFPE) samples were cut in 10-μm-thick sections using a paraffin sectioning method, and the frozen samples were cut into 20-μm-thick sections. When necessary, they underwent laser microdissection using Leica*CM1860*cryostat (Life Technologies, Carlsbad, CA) to enrich tumor cells. DNA was extracted using QIAamp DNA FFPE Tissue Kit (Qiagen, Hilden, Germany) or QIAamp DNA Mini Kit (Qiagen). This study was approved by Ethics Committee in Chiba University and Saitama Cancer Center.

### Histological evaluation

The FFPE specimens were cut in 4-μm thick sections and stained with hematoxylin and eosin. Two experienced pathologists performed histopathological examination, and the tumor cell content was confirmed to be > 70%. When the content was < 70%, laser microdissection was performed to enrich tumor cells. In this study, carcinoma *in situ* and invasive carcinoma were considered as cancer.

### Bisulfite treatment

Bisulfite conversion of 500 ng of genomic DNA from each tissue sample was performed using Zymo EZ DNA Methylation Kit (Zymo Research, Irvine, CA), and the DNA was eluted in 80 μL of 10 mEq Tris buffer. By bisulfite treatment, unmethylated cytosine is converted to uracil, i.e., recognized as thymine (T) after PCR reaction, but methylated cytosine is not converted, i.e. cytosine (C) after PCR reaction. Unmethylated DNA and methylated DNA are therefore distinguishable by detecting the difference of T and C in the sequence after bisulfite treatment.

Methylation control samples (0%, 25%, 50%, 75% and 100%) were prepared as previously described [12]. Briefly, human peripheral lymphocyte DNA was amplified using GenomiPhi v2 DNA amplification kit (GE Healthcare Life-Science, Buckinghamshire, England). The amplified DNA was not methylated in any CpG sites, and was used as unmethylated (0%) control. The amplified DNA was methylated by *Sss*I methylase and used as fully methylated (100%) control. Other methylation control samples (25%, 50%, and 75%) were prepared by mixing 0% and 100% samples at a ratio of 3:1, 1:1 and 1:3. These control samples were also treated with bisulfite in the same manner.

### Methylation analysis

Methylation levels were quantitatively analyzed by pyrosequencing on the PyroMark Q96 (Qiagen) using six Group-1 markers (*p16INKA*, *TIMP3*, *SPON1*, MINT17, *MLH1* and *CACNA1G*) and 14 Group-2 markers (*ADAMTS1*, *TMEFF2*, *STOX2*, *COLA4A2*, *EDIL3*, *UCHL1*, *RASSF2*, *ELMO1*, *PPP1R3C*, *PPP1R14A*, *BNIP3*, *ZNF447*, and *NEUROG1*) as previously reported [12, 28]. Primer sequences of the methylation markers are shown in supplementary Table 1. Briefly, the biotinylated PCR product was bound to Streptavidin Sepharose High Performance (Amersham Biosciences, Uppsala, Sweden), washed, and denatured using a 0.2 mol/L NaOH solution. After addition of 0.3 μmol/L sequencing primer to the single-stranded PCR product, pyrosequencing was carried out according to the manufacturer's instructions. By using methylation control samples, it was confirmed in each pyrosequencing assay that methylation analysis for the 20 markers was highly quantitative. Primer sequences and PCR conditions are available in our previous study [26].

### Mutation analysis

Mutations of *BRAF* (nucleotide 1799) and *KRAS* (nucleotide 34, 35, 37 and 38) were analyzed by genotyping assay on the MassARRAY platform as previously described [31]. PCR and extension primers for these mutations were previously designed using MassARRAY Assay Design 3.0 software (Sequenom, San Diego, CA) and applying default single base extension settings and default parameters. DNA was amplified by PCR and a single base extension reaction was performed using a custom mixture of nucleotides and extension primers that hybridized immediately adjacent to the mutations. Reaction products were transferred to a SpectroCHIP (Sequenom) and mass difference was analyzed using MALDI-TOF mass spectrometry to identify the extended base at the possible mutation site (Figure 1A).

*APC* mutations were analyzed by targeted sequencing of all 18 exons of *APC*. By using Ion AmpliSeq™ Library Kit 2.0 (Thermo Fisher Scientific, Waltham, MA) and Ion Xpress™ Barcode Adapter 1-16 Kit (Thermo Fisher Scientific), an amplicon library of the targeted exons was prepared with an Ion AmpliSeq™ Custom Panel (Solution ID: IAD45865_089, Thermo Fisher Scientific) designed with Ion AmpliSeq™ Designer (www.ampliseq.com) for 18 exons of the *APC* gene. The custom panel with 90 primer sets was prepared (Supplementary Table S2), and the coverage rate was 99.42%. After 100 ng of each genomic DNA sample was amplified using Ion AmpliSeq™ HiFi Master Mix (Thermo Fisher Scientific), amplicon libraries were constructed according to the manufacturers' instructions. After the emulsion PCR was carried out using the Ion OneTouch™2 System and Ion PGM™ Template OT2 200 Kit (Thermo Fisher Scientific), sequencing was performed with an Ion Torrent Personal Genome Machine (PGM) system using the Ion PGM™ Sequencing 200 Kit v2 and Ion 316™ Chip v2 (Thermo Fisher Scientific). The sequence data were processed with standard Ion Reporter™ Software (Thermo Fisher Scientific), a suite of bioinformatics tools, mapping to human genome sequence (build GRCh37/hg19).

### Immunohistochemistry

Immunostaining for TP53 was conducted using DO-7 anti-mouse monoclonal antibody (Santa Cruz Biotechnology, Santa Cruz, CA) as previously described [12] and samples with nuclear staining were considered TP53-IHC(+) and, thus, designated as *TP53*-mutation(+). Immunostaining for CTNNB1 was performed using anti-mouse monoclonal antibody (BD Transduction Laboratories) as previously described [28, 37]. Activation of the *WNT* signaling pathway, e.g., *APC* inactivation [38], resulted in the accumulation of CTNNB1 in the cytoplasm and/or nucleus. CTNNB1 activation was considered positive, (i) if nuclear staining was positive in at least one tumor cell per high-power field, or (ii) if cytoplasmic staining was positive in > 25% of tumor cells [39].

### Statistical analysis

Differences in methylation levels of each marker were analyzed using Student's *t*-test. Mutation status and tumor location were compared between intermediate- and low-methylation epigenotypes, or between adenoma and carcinoma using Fisher's exact test. These statistical analyses were performed using SPSS, ver. 11.0 (SPSS Inc., Chicago, IL). Unless otherwise specified, *P* values < 0.05 were considered to denote statistical significance. Unsupervised 2-way hierarchical clustering was carried out based on the City-block distance and the complete linkage-clustering algorithm using Cluster 3.0 software. The heatmap was drawn using Java Tree View software. Correlation of methylation level of each marker with *KRAS* mutation, tumor location, and age was evaluated by linear single regression of R software (https://www.r-project.org/).

## SUPPLEMENTARY MATERIALS FIGURES AND TABLES


