# Supplementary material for: Two subtypes of colorectal tumor with distinct molecular features in familial adenomatous polyposis
Source: Oncotarget. 2016 Aug 22;7(51):84003–16. doi: 10.18632/oncotarget.11510 (PMC5356641; doi:10.18632/oncotarget.11510)
Supplement: Supplementary file 1 [file oncotarget-07-84003-s001.pdf]

Two subtypes of colorectal tumor with distinct molecular features in familial adenomatous polyposis

Supplementary Materials include:

- Supplementary Table S1
- Supplementary Table S2
- Supplementary Fig. S1
- Supplementary Fig. S2
- Supplementary Fig. S3

Supplementary Table S1. Primers for pyrosequencing analysis of methylation marker genes

Genes	PCR Primers (Forward/Reverse)		Sequencing primers
**Group-1 markers**			
SPON1	**F**	AGGAGGTTGTTGTTTTAATTTTTAGTTATA	AGTTATATTTAGGATTTTTTGGAG
	**R***	AATTATTTCTCAAACTTTCCCTCCTCTA	
TIMP3	**F**	AGAGATATTTAGTGGTTTAGGTG	GGTGGGAGTGGGGTTA
	**R***	AAACCCTCCTACCCCTTCT	
CACNA1G	**F**	TGTTTTGGTTTAAGTAGAAGAAAAT	TGGTTTAAGTAGAAGAAAATTAT
	**R***	ACCCAAATCAACAAAAAAAAACCC	
MLH1	**F**	ATGTGGATGAAGTTTAAAAGAAGTAAGAT	ATGGAAGTAGAAGAGGTTTTAGTTT
	**R***	AAAACTCCACCACCAAATA	
MINT17	**F***	AAGTGGGAGAAGAGGAAGAGAAAAAATA	TATCCCTCCCCATCT
	**R**	CTATCCTCCCCAAACTTCT	
p16INKA	**F***	GGTGGGTAGAGGGTTTGTA	GGGAGTAGGGGATGG
	**R**	CCAATTCCCCTACAAACTT	
**Group-2 markers**			
ADAMTS1	**F**	GTTTTTTGGGGTTTTAATGTAG	TTAATGTAGAGAGTTGTGTT
	**R***	TTCCCCATATCCCTACCCAACTTAC	
BNIP3	**F**	AGAAGTAGATTTATTTTTAGGTGGAATT	ATTTTTAGGTGGAATTTTAGT
	**R***	AAAAAACCCCCATTCTCCAACT	
COL4A2	**F**	TTTTTTGTTTGGGAGTAGAAAGG	GGGGAGGGTGGGAGA
	**R***	AACCCCTATAAATACTCCTTAACC	
EDIL3	**F**	GGGATTTTTAGTTTATTTTTTATTTAGTTG	TTAGTTTATTTTTTATTTAGTTGTT
	**R***	ACTCAACCTCCATATCCCCCAATT	
EFEMP1	**F***	TTGGGAAGTTGAGTAGTTTTAGGG	AAATCCCCTTTCTTAACA
	**R**	ACCCCACAAAAAAATAAAATCCCCTTTCTT	
ELMO1	**F**	GGAGGAAGGAAGAGGAAGTGA	GAGGAAGTGAGAGTAG
	**R***	CCAACACACCCACTTACACTCTAAAT	
PPP1R3C	**F**	GTGTTTGGGAGTAGATAAG	TTTTTAGGAGTAGGGTTTTTAGTTT
	**R***	CACAACTCCAAACCTTACC	
PPP1R14A	**F**	GGGGGTTTGGGATAGATAT	GGGATTAGGTTTGTTTG
	**R***	CTCCCCCACCAACAACCC	
RASSF2	**F**	AGGGTTGTTAGGTTTTTTTTTAGT	GTTAGGTTTTTTTTAGTTTTGTA
	**R***	CCCATCCCCCAAATCTCTAAAACTT	
STOX2	**F**	AGGTTGGGGTAGTTGTTAAG	GGGTAGTTGTTAAGGTTT
	**R***	TCCCATCAAACTTCTCATTTTCA	
TMEFF2	**F**	ATTTAGGGATTGGGTTTAGT	AGGGTAGTTAGTTGAGAAGT
	**R***	CCCTCCTTATAACAACAACT	
UCHL1	**F***	GGTAGGGTTTTTAAATTTTGTAGTTTTATT	CCACCAAATTATCTCACC
	**R**	CCACAACCACCAAATTATCT	
ZNF447	**F**	GGGGTAGTTGAGTAGTAGGTGG	GGTAGGTTTAGGGGATGTAG
	**R***	CCTCACCCTCTACCCTATTAAAATC	
NEUROG1	**F**	AGTTTGGGGTTGTTATTTTGTGTTA	GTTGTTATTTTGTGTTAGTTG
	**R***	AAAAAACCAAACCAAATTCTCC	

* Primers with 5′-biotin tag.

Supplementary Table S2. Primer sets for targeted exon sequencing of *APC* mutation

	Amplicon_ID	Ion_AmpliSeq_Fwd_Primer	Ion_AmpliSeq_Rev_Primer
1	AMPL393460395	GGCTTCAAGTTGTCTTTTTAATGATCCT	GTACCTGCTGTAAGTCTTCACTTTCA
2	AMPL393647274	TGAAGATGACCTGTTGCAGGAAT	ACCTTCCTGAATAGCTTTCCAATCAAA
3	AMPL393703418	AGACCTGTATTAGTACGCCAGTCA	AGAGACATATCAGGAAGGGAAGGAC
4	AMPL394027070	CCTTATAGGTCCAAGGGTAGCCA	TGCCTCAGTTTCCAGTTTTGTAAGAT
5	AMPL396737537	CCAAAGTCCTAAGCGCCATTC	GACCAGTGAAGACAAAGTATACTATCAAA
6	AMPL423994143	AACACTGATTACTTCATCCTGGAAAGG	TGAGTACCAAGCATTGACAACAATGA
7	AMPL1073833676	GTCATCAGCTGAAGATGAAATAGGATGT	CTGCAGTCTGCTGGATTTGGT
8	AMPL1381214544	CTCATGCACCATGACTGACGTA	CTGATTTGCCTTGCTTCATATTCCAAT
9	AMPL1481686998	TCCTGTTTATACTGAGAGCACTGATGAT	TCTCTTCTTCTTCATGCTGTTCTTCTTC
10	AMPL3659103069	AGAGGGTGACTGATGATACATGCATA	CCTGGAAGTTATAAACACAGGTAAGGG
11	AMPL4146798162	CCATTATTGAAAGTGGAGGTGGGATAT	TAGGTCTTTTTGAGAGTATGAATTCTGTAC
12	AMPL4169531152	GTGTTCTTTTTAACAGGAAGTACTTAAACAACT	CGAGAGAAGCTGTACTTGGATCTAC
13	AMPL4536989699	CGAAGAATAGCCAGAATTCAGCAA	ACATGTTTTCTTTTCTCAGAATAACTACCTA
14	AMPL4738848087	GAATTTGTCTTGGCGAGCAGAT	AGGAAATCTCATGGCTAAAAGAAGG
15	AMPL5138766960	GAAGATAGAGTCAGAGGAAGTTTTGCT	CTGATTGTTGGTTGGAGGTTAGTTC
16	AMPL5138803846	CTCGAGGCAGGACAATGATTCATA	GGGTCGAATCTCTAGATCCTGAT
17	AMPL5138945736	GCACCTACTGCTGAAAAGAGAGA	TGTTTCATTCCCATTGTCATTTTCCTG
18	AMPL5138987769	GTGTCAGCCATTCATACCTCTCAG	AATCGAGGGTTTCATTTGACCTCTT
19	AMPL5189920641	GCATCCCATCCCAACTTCCTTTAATTATT	AAGCAGGCTGGGTAAACTTGT
20	AMPL5189967645	CCACAAATAGTACTTCTCAGACCGTTTC	TTGAATCTTTAATGTTTGGATTTGCCTT
21	AMPL5196704502	GGAGAGAGAAGCAGCTGTGTAAT	CCAAGGAAAGGCCATCGGTTTA
22	AMPL5665143056	GCAACCCCAAAAGCATGTTAGT	TTCCTCCTTGAGCCTCATCTGTA
23	AMPL5665206447	AGGAAGCATTATGGGACATGGG	AAAGTTTCTGATAAGTGCTGAGCATCT
24	AMPL5692057662	CTCCACTGGAGCTCAGTAAAAGTAAAT	CCCTAACATACAGGGTGATAAAAAGTGT
25	AMPL5692124014	CATTGCGCCTGCGCATAA	CTTCCTCACCAACAGCCAAC
26	AMPL5693252847	TTTGCATGTACTGATGTTAACTCCATCT	GCTTCTGGAAATATGCATTCAGGACTAA
27	AMPL5693677962	GGGTTCATTTCCAAGAAGAGGGT	AAGTGCAATCAATAACATGCTATCTTTG
28	AMPL5693708974	TGATTGTCTTTTTCCTCTTGCCCTTT	GCTACATCTCCAAAAGTCAAGTTTGTCA
29	AMPL5763999285	AGAGACTTAGTCAAGGGCAGATGA	CAGCAGGACAGATCTGATGTTCAA
30	AMPL5957526178	TGTGCTCCAAACAAAACAAAAATCTGT	CAAATGTGAAACAAGACCCTTCAAACAA
31	AMPL393540352	GATGCTGTTCCCAGGTACTGTT	CCCGAGAACTGAGGGTGGTA
32	AMPL395267986	ACCTGGTGCCTTGAAAATCACAT	AAGGTCAATCATTTCAAAGATAGTAAATTGAAG
33	AMPL396044271	AGCACTTTAGGTAGAGAAGTTTGCAAT	AGCGTAATACCAGTCTTTTTCCTTTTCT

34	AMPL434747307	CAGAGGTTAGTAAATTGCCTTTCTTGTT	CTGCCTAAAAGTTAGATAAAATCAAAATAGTCC
35	AMPL674537212	CTTCGACAAGAGCTAGAAGATAATTCCA	AAGTGTCTTACCTCAAGTTTACAAGAG
36	AMPL1076312570	CAGTGTCACAGCACCCTAGA	TCAAAACTATCAAGTGAACTGACAGAAG
37	AMPL1481653367	CTCCTTATTTTTACCCTGACCCAAGT	GGGACATTTTTGACCGCAGTTTT
38	AMPL1481656535	ATTTTATTTTTCAGTGCCAGCTCCTG	ACAGAAGTTGGTGGCCTTATATCCTAA
39	AMPL1481668620	CTTACAAACAGATATGACCAGAAGGCA	GAGCCAAAATAAACACAGCCTTATGT
40	AMPL1481727237	TCTTGTTAGAGGGTTTTTGTTCTGGAA	TGTAAAACATACTGTACAAACATACTTGGCT
41	AMPL3659066251	AAATTCACAGTAATATGGTTCCCGATGA	ACAGATTTTTGTTTTGTTTGGAGCACAA
42	AMPL3751348285	GATATTACGGAATGTGTCCAGCTTGATA	ATAAAACATTGCTTACAATTAGGTCTTTTTGA
43	AMPL4042686722	TTCTATTCTTACTGCTAGCATTAAAAACAAAAA	TGCTCGCCAAGACAAATTCCT
44	AMPL5138551581	GTGGGTCAAGTAAAGCACCTTCTA	ACCTGTTTGTTTGGTAAGGTTCTGT
45	AMPL5138676396	GATACCATTCCTACAGAAGGCAGAAG	TGGTGAAGTTGGTTTCTTTTTCTTACCA
46	AMPL5138696684	CATGGTCTATCCCCTGATTCAGAAAAT	CCAATGTACTTTTCTCCCCTGGTTTT
47	AMPL5138764053	GCCTACCAATTATAGTGAACGTTACTCT	CATTAGATGAAGGTGTGGACGTATTCTC
48	AMPL5138854074	GGCTAAAGTTACCAGCCACACA	GGTCAATGTCACTGAGAGAACTCA
49	AMPL5139022082	TGTGGAATTAAGAATAATGCCTCCAGTT	CTTGCCACAGGTGGAGGTAAT
50	AMPL5189652396	CTGGTATTTGAGGTGAGATGGCT	GTGGCAACAGTCTTCTTCAATTGTTTT
51	AMPL5189763000	CCGGTGATTGACAGTGTTTCAGAA	GTTTGCTTGAGCTGCTAGAACTG
52	AMPL5189869306	CCAGGCACAAACTCCAGTTTTAA	GTGCTTACTCGAGGAAGGGATG
53	AMPL5269795675	TGTCAGTAGTAGTGATGGTTATGGTAA	TTCATTCTGTGAAGGACTTTGCCT
54	AMPL5269851832	TGTTTATCATACAGACACTTCATTTGGA	AATGTCTTAGCAAAGTAGTCATGGCA
55	AMPL5665324055	CCCACAGGTGGAAATGGTGTAT	CTTGTCATCAGGCTGTGAGTGAA
56	AMPL5665328258	GAAACAAAAAGCCCTAGAAGCAGAATT	CAGAACGAGAACTATCTAAGCTTCCTC
57	AMPL5691799956	GAGGACAACAAAGAATGGAGCATA	CTTAGATGTTTTACAAAAGCAGGCACA
58	AMPL5691890571	TTGCAACTGGGTCTGACATGA	AAGGAAGACTTCCCTCCACTGTATAA
59	AMPL5693711929	CAGTGAGGGACGGGCAATAG	CTCCGGTAAGTAAGAGTGCCAA
60	AMPL5693742773	CTAAGACGATATGCTGGAATGGCTT	GCGAATGTGAAGCACAGGTTTT
61	AMPL395034078	ACATGATGTGCATAGAGATAGCTACAGT	TCATGTCTCATTAATAAAAGAGCAGCCA
62	AMPL674483179	AGACTCCACTGTTTCATCCTCTTAGA	CATATGAAGCTGCAGCCATCCT
63	AMPL675154644	GTTTGACAAAGGAAGAACAGATAGCAAA	CATAGAGCATAGCGTAGCCTGTAAATTA
64	AMPL675649764	AAGGGACACACTTCACTTTCCC	GCTTGTTCTGAGATGACCTCTGTTA
65	AMPL707909899	GAGGCACTCTTGATGGTTAGGAAAA	TCTATCTGTAGTGTTCATTATTTTAAGACAAGC
66	AMPL1481706928	TGCTTCTTGCTGATCTTGACAAAGA	CAGGCCTAAAGTTGGGTAAAACATATTG
67	AMPL1485024388	GGGTGGAGAAACTGGCATAAAA	TGGCCTGTAGTCCCCCTAATTTAA
68	AMPL3897592468	TGATTAATTTGCAGGTTATTGCGAGTG	AGGTACCTTTTTAACTTCTAAAGCACAT
69	AMPL4169443268	TGCATTAAGAGTAAAATTCCTCTTACTGT	GCTGCTTGCATTGCTATTACATGA

70	AMPL4169525311	GGTGCGTGCTTTGAGAGTGA	CCATAGCTTCATCTTCAATACTTCCTTGT
71	AMPL4536546825	AGAATGATTTGACATAACCCTGAGCTT	CGTATACGAAGTATGTCCTTTTCGAT
72	AMPL4536993154	TCTGGGAACCAAGGTAACAGAAGA	TGCCACCACACTGGCTAAA
73	AMPL5138555689	CGGTTTGCTTTTCTCATAATTCCTCTC	TTTTTGGCATTGCGGAGCTTATAC
74	AMPL5138683853	TCCAGGTAGACAGATGAGCCA	GCTTGGAGCTTCTTTGATGAAAGT
75	AMPL5138742097	TCTGCACCCAACAAAAATCAGTTAGA	GCGTGTAATGATGAGGTGAATCAAA
76	AMPL5138765656	ACAAGTAATAAAGGCCCACGAATTCT	GGAGCTATTTCGAACTCCTGGAA
77	AMPL5138795614	CCCCACTCATGTTTAGCAGATGTA	GCAGCTTGCTTAGGTCCACTC
78	AMPL5138822647	AACCGAACATATGTCTTCAAGCAGT	CTGCTTCCTGTGTCGTCTGATT
79	AMPL5189736613	ACTTTATTTGTGGTAGGTACAGTTCTGG	AGTGCCCCACCATGCTTAAAA
80	AMPL5189852444	AGTTCTATAGTGGAACGTACCCCATT	CAGATGTCACAAGGTAAGACCCA
81	AMPL5189937520	CGTGAGCACAGCAAACATTCAT	AGCACCATTTGTAGCACCTGAG
82	AMPL5238387025	TGGTACAATCATATTATGCCTTTTGTCT	AATGAGGTTCTTGAGCATGCTAACT
83	AMPL5269967340	CAGATGAGCAGTTGAACTCTGGA	TGTGGTTGGAACTTGAGGTGTTT
84	AMPL5665412864	TGCAGCACTCCACAACATCA	TTTCTCCTCTTAGTCATAAAGACAGCAT
85	AMPL5665416512	GTTACCCAGCTCCTCTTCATCAA	GGTAGACCCAGAACTTCTGTCTTC
86	AMPL5665459931	CCAGCCCAGACTGCTTCAAA	GCATATCATCCCCCGGTGTAAA
87	AMPL5693644155	ACTTAGATAGCAGTAATTTCCCTGGAGT	CCAGTACTTTCTCTGCTTCCATTTACAA
88	AMPL5693806435	AGATGGTGCACTTGCATTTTTGG	CCTGTGGTCCTCATTTGTAGCTAT
89	AMPL5694593983	CCCTTTGCCCGCTTCTG	GGGTGAGACATGGAGAGAAGAGA
90	AMPL6417171619	CTCCAATGCCTGTACTGTGTCT	CATAGAAGTACTTGAAGTGGTTTGCAAC

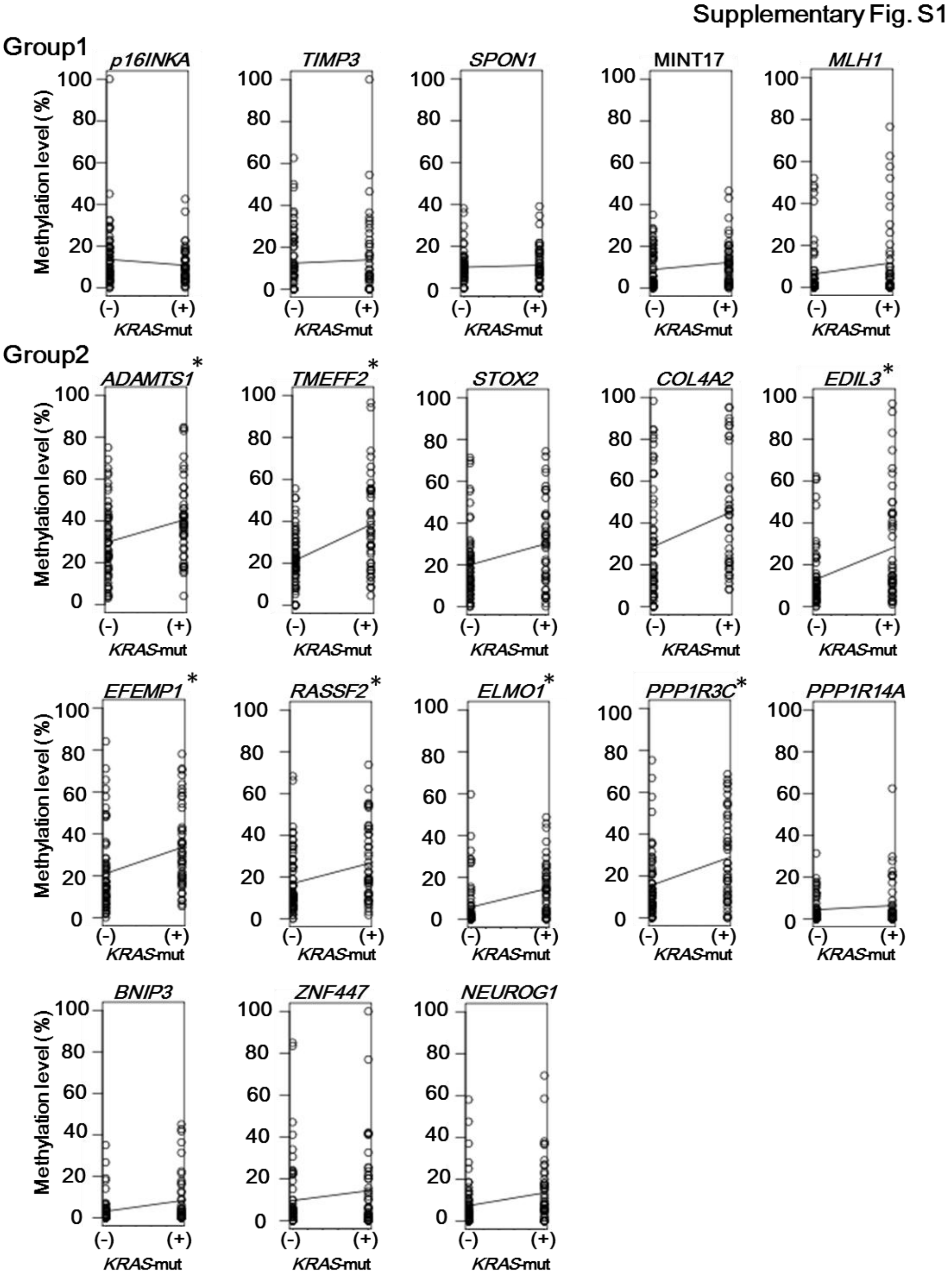

Supplementary Fig. S1.

Comparison between methylation levels and *KRAS*-mutation using linear single regression model. Eight of 14 Group-2 markers (*ADAMTS1*, *TMEFF2*, *EDIL3*, *EFEMP1*, *RASSF2*, *ELMO1*, and *PPP1R3C*) showed significant correlation between higher methylation level and *KRAS*-mutation(+) status (**P*<0.004), while none of Group-1 markers did. Since 14 Group-2 markers were evaluated, *P*-values <0.004 (i.e. 0.05/14) were considered significant.

Supplementary Fig. S2

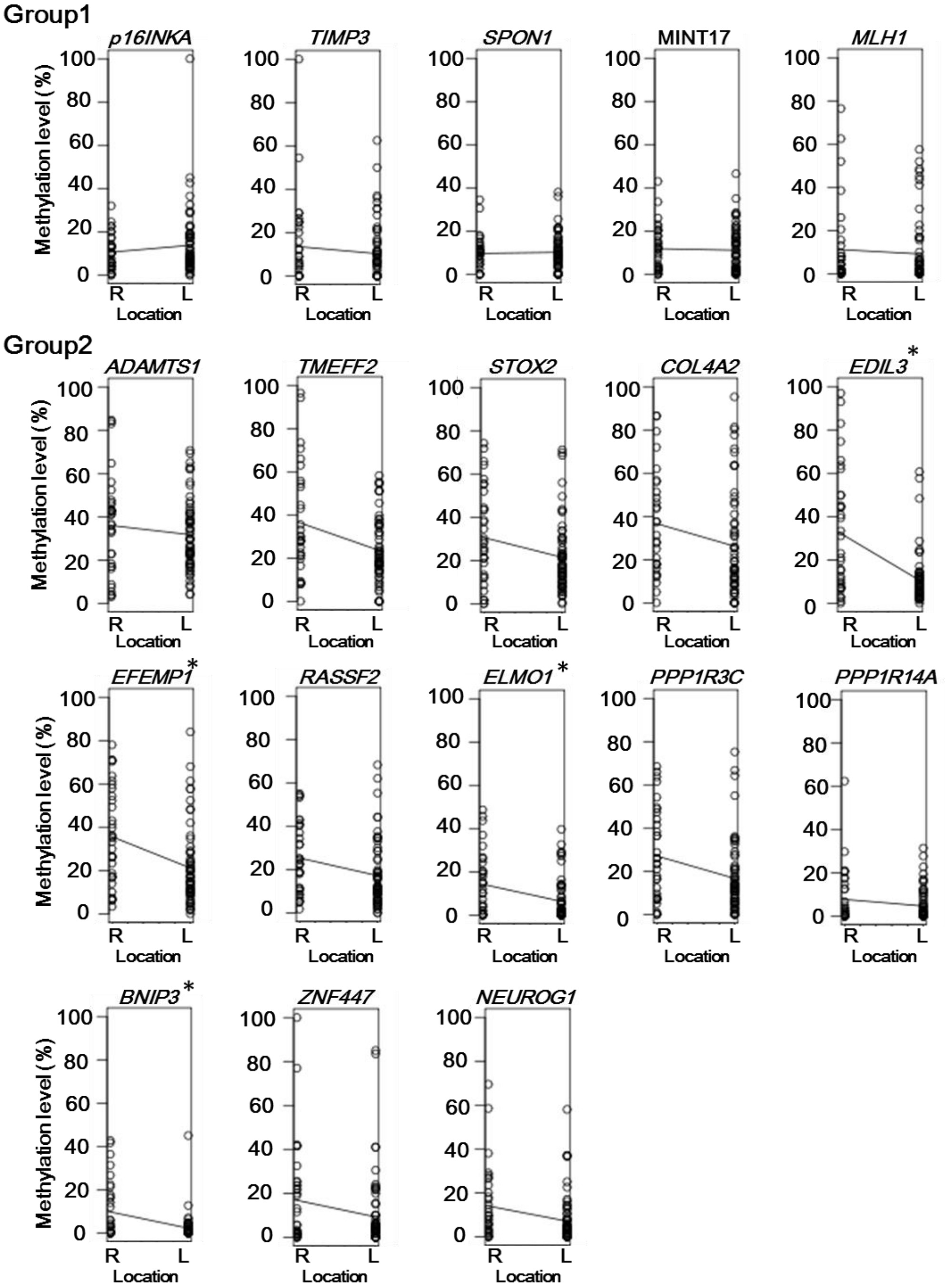

Supplementary Fig. S2.

Comparison between methylation levels and tumor location using linear single regression model. Five of 14 Group-2 markers (*EDIL3*, *EFEMP1*, *UCHL1*, *ELMO1*, and *BNIP3*) showed significant correlation between higher methylation level and proximal location (*P<0.004), while none of Group-1 markers did. Since 14 Group-2 markers were evaluated, P-values <0.004 (i.e., 0.05/14) were considered significant.

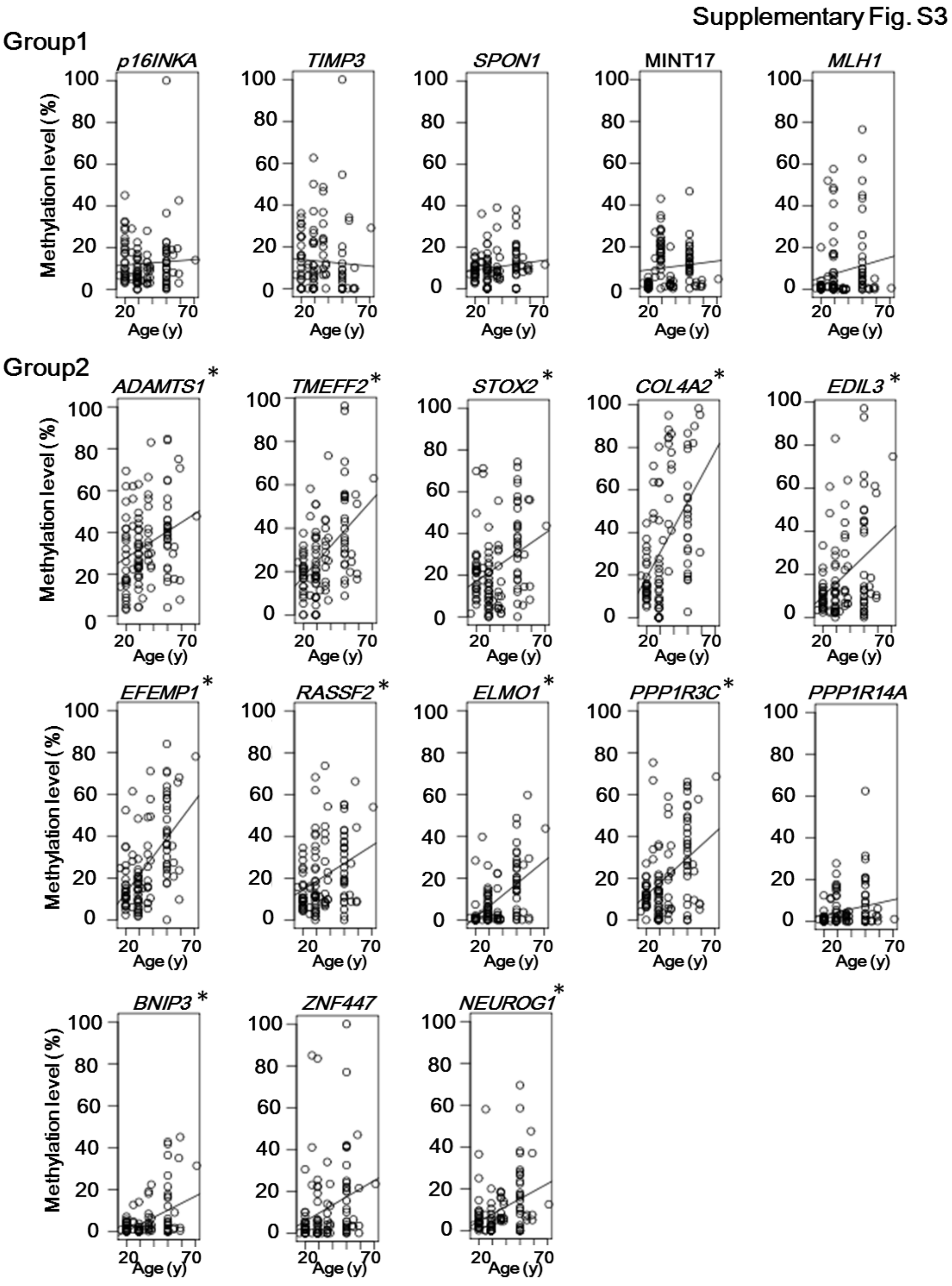

Supplementary Fig. S3.

Comparison between methylation levels and age using linear single regression model. Twelve of 14 Group-2 markers (*ADAMTS1*, *TMEFF2*, *STOX2*, *COL4A2*, *EDIL3*, *EFEMP1*, *RASSF2*, *ELMO1*, *PPP1R3C*, *BNIP3*, and *NEUROG1*) showed a significant correlation between higher methylation level and age (*P<0.004), while none of Group-1 markers did. Since 14 Group-2 markers were evaluated, P-values <0.004 (i.e., 0.05/14) were considered to be significant.
